# Association Analysis in Rice: From Application to Utilization

**DOI:** 10.3389/fpls.2016.01202

**Published:** 2016-08-17

**Authors:** Peng Zhang, Kaizhen Zhong, Muhammad Qasim Shahid, Hanhua Tong

**Affiliations:** ^1^State Key Laboratory of Rice Biology, China National Rice Research InstituteHangzhou, China; ^2^State Key Laboratory for Conservation and Utilization of Subtropical Agro-Bioresources, South China Agricultural UniversityGuangzhou, China

**Keywords:** association analysis, genotyping, linkage disequilibrium, marker density, phenotyping, population structure, *Oryza sativa*

## Abstract

Association analysis based on linkage disequilibrium (LD) is an efficient way to dissect complex traits and to identify gene functions in rice. Although association analysis is an effective way to construct fine maps for quantitative traits, there are a few issues which need to be addressed. In this review, we will first summarize type, structure, and LD level of populations used for association analysis of rice, and then discuss the genotyping methods and statistical approaches used for association analysis in rice. Moreover, we will review current shortcomings and benefits of association analysis as well as specific types of future research to overcome these shortcomings. Furthermore, we will analyze the reasons for the underutilization of the results within association analysis in rice breeding.

## Introduction

Rice (*Oryza sativa* L.) is one of the most important components of the human diet in many regions of the world and feeds more than 50% of the world's population. Thus, increasing the yield of rice through genetic improvement is important to meet the food demands of a growing global population. After the completion of the rice genome, extensive genetic studies have been conducted to characterize the biological functions for hundreds of rice genes.

In the early Twentieth century, Jennings (1917) raised the concept of linkage disequilibrium (LD), which refers to the non-random combinations among different genetic markers. The main mechanism of LD existence in a population over time is the association between alleles at different loci. Hence, there is a possibility of detecting quantitative trait loci (QTL) by estimating LD between loci and potential QTLs. The tightly linked loci that have significant correlation with QTLs can be detected through genetic markers or loci distributed in the genome or those nearby the candidate genes. Association analysis (AA) based on LD can overcome the limitations of linkage mapping (i.e., we can detect only two alleles at any given locus in a bi-parental cross and a low mapping resolution are major limitations of linkage mapping; Flint-Garcia et al., 2003). Yu and Buckler (2006) compared AA with other linkage mapping methods in detail, and revealed that AA could explore most of the recombination events and mutations in a given population with higher resolution. However, linkage mapping methods are best suited for populations with low genetic diversity (Flint-Garcia et al., 2005).

In general, linkage mapping is a conventional method for gene mining in rice. To identify QTLs by linkage mapping, the development of one or several segregating populations through crossing of two lines/varieties is required (e.g., Recombinant Inbred Lines-RILs, F_2_, Double Haploid and Backcross populations). Therefore, the accuracy of QTL detection largely depends on the selected lines (Zhang Y. M. et al., 2005). AA can incorporate a relatively large portion of natural variation in a species and localize associations to much smaller genomic regions, because sampling diversity incorporates many more recombination events than traditional recombinant inbred lines (Nordborg and Weigel, 2008). AA has two major advantages compared with linkage mapping: (1) wider genetic variation, and (2) higher mapping resolution (Remington et al., 2001). AA has been widely applied in the form of genome-wide association studies (GWAS) and candidate-gene association studies (CGAS). GWAS typically focus on associations between single-nucleotide polymorphisms (SNPs) and major traits, whereas CGAS analyzes specific variants of a particular gene often selected on the basis of a biological hypothesis. Moreover, GWAS is often utilized when we are interested in finding out all the genomic regions that may control a specific trait. If the information about the genetics of a target trait is available and CGAS can be predicted on the basis of available information, we can confirm the genes that control the trait of interest. Caldwell et al. (2006) defined AA as “two tiered” in a study on barley, in which a lower resolution AA based on genome wide scanning was used to detect the candidate region in elite materials, and a higher resolution AA was carried out to mine candidate genes in landraces and wild accessions. The power of AA is largely determined by population size, the genotype relative risk (only for human diseases), effect size, marker density, LD decay rate between marker and target allele as well as errors in phenotypic and genotypic data, and the desired statistical significance level (Gordon and Finch, 2005).

In plants, AA has been applied efficiently to dissect many complex quantitative and qualitative traits under biotic and abiotic stresses in diploid and polyploid plants (rape seed and bread wheat; Harper et al., 2012; Ling et al., 2013). Large-scale GWAS have led to the discovery of thousands genetic signals across the plant genome associated with the quantitative traits of plants. Moreover, it has been demonstrated that new genes can be effectively identified on the basis of GWAS data (Si et al., 2016).

Although AA is an effective way in which we can construct fine maps for quantitative traits, there are a few issues which need to be addressed. For instance how to (1) eliminate the influence of linkage, population structure, and familial relatedness to reduce the false positives in AA; (2) reduce the false negatives that can lead to overcompensating for population structure and relatedness in AA based on a mixed linear model (MLM) or other models, and limited power to detect LD among the populations with lower genetic diversity (i.e., low frequency of rare alleles and genetic variants); (3) improve the computational capacity of a model for AA; (4) improve the repeatability of significant trait-marker associations in AA; and (5) utilize AA in rice breeding.

A review about rice AA will be very useful for AA applications in other plants because rice is considered as a monocot cereal model plant. In this review, we first describe the population type, population structure, LD level and the genotyping methods and statistical approaches used for rice AA. Then, we review the benefits of association analysis, shortcomings, and the possible types of future research to overcome these shortcomings. Furthermore, we also discuss the reasons for the underutilization of AA in rice breeding.

## Population types in rice AA

GWAS in crops usually requires a permanent resource—a population of diverse (and preferably homozygous) landraces or cultivars that could be re-phenotyped for many traits (Huang and Han, 2014). Rice landraces and cultivars selected from different germplasm resources were used in previous rice AA (Table 1). Rice landraces contain greater genetic diversity than cultivars and represent an intermediate stage in domestication between wild and elite cultivars (Londo et al., 2006). Mining the elite genes within these rice landrace is of particular importance for the genetic improvement of cultivated rice. Rice landraces have more elite genes or variations for biotic stress, abiotic stress, high-quality, and yield than varieties. Here, we recommend that the sampling population (e.g., core collection, mini core collection) should be created from rice landraces to use for rice AA.

**Table 1:** **Summary of association analysis in rice**.

**Association strategy**	**Sample size**	**Population**	**No. of subgroups**	**Significant level (*p*-value)**	**Genotyping method**	**Association method^①^**	**Trait**	**References**
CGAS	105	Landraces	–	–	Five sequencing markers based on *Wx* gene	Nucleotide diversity measure	Glutinous traits	Olsen and Purugganan, 2002
GWAS	218	Lines from US and Asia	–	<10^−3^	66 SSRs and 114 RFLPs	Discriminant analysis	12 agronomic traits	Zhang N. et al., 2005
GWAS	103	USDA germplasm	7	<5 × 10^−2^	123 SSRs	MLM	5 yield traits	Agrama et al., 2007
GWAS	90	USDA mini-core collection	3	<10^−3^	108 SSRs and 1 InDel	MLM	Stigma and spikelet characteristics	Yan et al., 2009
GWAS	170	Diverse landraces and cultivars	2	<10^−4^	126 SSRs and 6 InDels	MLM	6 morphological traits of Cheng's index and 3 agronomic traits	Wen et al., 2009
GWAS	84	Landraces	4	<5 × 10^−2^	24 SSRs	MLM	Amino acid contents	Zhao et al., 2009
CGAS	70	Diverse varieties	2	<5 × 10^−2^	Starch synthesis related genes in rice	MLM	Amylose content, gel consistency, and gelatinization temperature	Tian et al., 2009
GWAS	293	Landraces and cultivars	–	<10^−3^	179 RFLPs	Elliptic Fourier analysis	Grain shape	Iwata et al., 2010
GWAS	416	Landraces, cultivars, and breeding lines	7	<10^−3^	100 SSRs	GLM+Q	Starch quality traits	Jin et al., 2010
GWAS	192	Elite lines and varieties	–	<5 × 10^−2^	97 SSRs	MLM	Apparent amylose content, heading date, and head rice	Ordonez et al., 2010
GWAS	517	Landraces	2	<10^−7^	3,600,000 SNPs	LR–Q and MLM	14 agronomic traits	Huang et al., 2010
CGAS	303	Cultivars	2	<5 × 10^−2^	24 SSRs	LR+Q	Awn	Hu et al., 2010
CGAS	118	Glutinous accessions	2	<5 × 10^−2^	43 gene-specific molecular markers based on 17 starch synthesis-related genes	MLM	Rapid visco analyzer profile parameters	Yan et al., 2011
GWAS	416	Landraces, cultivars, and breeding lines	7	<5 × 10^−2^	100 SSRs	GLM+Q and MLM	Grain color, phenolic content, flavonoid content, and antioxidant capacity	Shao et al., 2011
GWAS	217	USDA mini-core collection	5	<6.45 × 10^−4^	154 SSRs and 1 InDel	MLM	14 agronomic traits	Li X. et al., 2011
GWAS	174	USDA mini-core collection	5	<10^−2^	156 SSRs, 2 InDels and 6 SNPs	GLM+Q and MLM	Silica concentration in hull	Bryant et al., 2011
GWAS	180	European Rice Core collection	3	<10^−2^	124 SNPs and 52 SSRs	GLM+Q, MLM–Q and MLM	Salt tolerant	Ahmadi et al., 2011
CGAS	346	Cultivated and wild rice	6	<5 × 10^−2^	2 sequencing markers based on *SsIIa* gene	GLM+Q and nested clade analysis	Starch quality	Yu et al., 2011
GWAS	383	Diverse landraces and cultivars	5	<10^−4^	44,000 SNPs	LR+Q, principle component analysis, and GLM+Q	Relative root growth in aluminum toxic	Famoso et al., 2011
GWAS	413	Diverse landraces and cultivars	5	<10^−4^	44,100 SNPs	SLM−Q, LR−Q and MLM	34 traits including agronomic, quality, and biotic stress	Zhao et al., 2011
GWAS	203	USDA mini-core collection	5	<6.45 × 10^−4^	154 SSRs and 1 InDel	MLM	14 agronomic traits	Li et al., 2012
GWAS	217	USDA mini-core collection	5	<5 × 10^−2^	154 SSRs and 1 InDel	MLM	Sheath blight resistance	Jia et al., 2012
CGAS	104	Diverse Landraces and cultivars	3	<5 × 10^−2^	8 sequencing markers based on *Ghd7* gene	GLM+Q	Plant height, heading date, and spikelets per panicle	Lu et al., 2012
GWAS	950	Worldwide varieties	5	<10^−7^	4,109,366 SNPs	LR–Q and MLM	Flowering time and with 10 grain-related traits	Huang et al., 2012b
GWAS	167	*japonica* accessions	6	<5 × 10^−4^	9727 DArT markers and 6717 SNPs	GLM+Q and MLM	Root traits	Courtois et al., 2013
GWAS	50	Waxy rice accessions	2		455 AFLPs and ISSR	MLM	Starch physicochemical properties	Xu et al., 2013
GWAS	529	Landraces and elite varieties	2	<5 × 10^−2^ <10^−7^	6,400,000 SNPs	LR and LMM	840 metabolic traits	Chen et al., 2014
GWAS	150	Landraces	2		274 SSRs	GLM and MLM	12 agronomic traits	Zhang et al., 2014
GWAS	366	*Indica* landraces	–	<5 × 10^−2^	800,000 SNPs	EMMAX	Blast resistance for 16 strains	Wang C. et al., 2014
GWAS	529	Landraces and elite varieties	–	<10^−8^ < 10^−6^	4,358,600 SNPs	FaST-LMM	13 traditional agronomic traits and 2 newly defined traits during the rice growth period	Yang W. et al., 2014
GWAS	126	High-yielding or primary ancestral cultivars	4	<10^−4^	1152 SNPs	MLM	6 yield traits	Yonemaru et al., 2014
GWAS	270	Landraces	2	<5 × 10^−4^	241 DArT markers and 25,971 SNPs 262 SSRs	MLM	Flowering time	Phung et al., 2014
GWAS	540	Landraces	7	<10^−2^	262 SSRs	GLM	Seed vigor (root length, shoot length, and shoot dry weight)	Dang et al., 2014
GWAS	100	Landraces and cultivars	3	<10^−2^	81 molecular markers	MLM	15 morphological traits	Jahani et al., 2014
GWAS	300	Cultivars	4	<10^−4^	369,000 SNPs	MLM	Grain concentrations of arsenic, copper, molybdenum, and zinc	Norton et al., 2014
GWAS	220	Landraces and cultivars	3	<10^−5^	4929 SNPs	CMLM	Salinity tolerance	Kumar et al., 2015
GWAS	328	Cultivars	5	<10^−4^	30,000 SNPs	MLM	Ozone tolerance	Ueda et al., 2015
GWAS	363	Elite breeding lines	4	<5 × 10^−6^	71,170 SNPs	LMM	19 agronomic traits	Begum et al., 2015
GWAS	95	Landraces and cultivars	7	<5 × 10^−2^	263 SSRs	GLM	Grain-filling rate	Liu E. et al., 2015
GWAS	175	Japanese rice collection	–	<10^−5^	3168 SNPs	EMMA	Metabolites	Matsuda et al., 2015
GWAS	1495	Elite hybrid rice varieties	2	<10^−6^	1,654,030 SNPs	EMMAX	38 agronomic traits	Huang et al., 2015
CGAS	529	Chinese core collection and world core collection	5	<10^−3^	41 CCT genes based on *GHD7*	GLM+Q	Heading date	Zhang et al., 2015
GWAS	176	Japanese *japonica* varieties	0	<10^−5^	43,323 SNPs	GLM+K	Agronomic traits	Yano et al., 2016

*GWAS, Genome-wide association study; CGAS, Candidate-gene association study*.

① *+, considering population structure; −, not considering population structure; GLM, generalized linear model; LR, logistic regression; MLM, mixed linear model (Q+K model); SLM, simple linear model; LMM, linear mixed model. Q, population structure; EMMAX, efficient mixed-model association eXpedited; FaST-LMM, factored spectrally transformed linear mixed model; CMLM, compressed mixed linear model; EMMA, efficient mixed-model association*.

It is very hard to mine and utilize the exotic genes existing in rice accessions (i.e., 7.75 × 10^5^) in the world (FAO, 2010) by either linkage mapping or AA. The maximum population size used for rice AA was 1495 rice accessions in a previous study (Huang et al., 2015). One of the methods to utilize the huge germplasm with AA is to construct the core collection. A core collection is a subset chosen to represent the abundant genetic diversity of a collection with a minimum of redundancies (Frankel, 1984; Frankel and Brown, 1984a,b). Construction of core collection has been widely applied in rice as well as in other crops (Yu et al., 2003; Liu W. et al., 2015). A rice core collection consisting of 150 accessions based on 48 morphological traits from 2262 accessions of Ting's collection has been constructed and used in AA (Li X. L. et al., 2011; Zhang et al., 2011, 2014). The abundant genetic variations in the rice core collection provide an important reservoir of genetic diversity and potential sources of beneficial alleles for rice breeding. Furthermore, the United States Department of Agriculture (USDA) also constructed different mini-core collections and core collections of rice with different sampling sizes, and AA was conducted within these collections (Yan et al., 2009; Bryant et al., 2011; Li X. L. et al., 2011; Li et al., 2012; Jia et al., 2012). Moreover, a salt tolerant European rice core collection consisting of 180 accessions was constructed for AA (Ahmadi et al., 2011).

In addition, family-based populations can also be used for AA and improve the power of AA because there is less population stratification in family-based populations (Gupta et al., 2014). For instance, nested association mapping (NAM) population, consisting of 25 RILs, was successfully created by crossing a diverse range of 25 important breeding lines with one common and well-characterized parent through AA in maize (Yu et al., 2008). Moreover, the multi-parent advanced generation intercross (MAGIC) populations, originally proposed for animals AA (Mott et al., 2000) and later used in *Arabidopsis thaliana* (Kover et al., 2009) and *Zea mays* (Chintamanani et al., 2010), were developed. The NAM and MAGIC populations have abundant recombinations or variants for gene identification. These populations could be used for rice AA and it could increase the detection power of traditional association mapping to detect rare alleles. A list of software that can be used for family-based AA is freely available (Ott et al., 2011).

The populations used for AA should possess as many phenotypes as possible (Flint-Garcia et al., 2005). The choice of appropriate germplasm resources to maximize the number of historical recombination and mutation events (and thus reduce LD) within and around the gene of interest is critical for the success of AA (Long and Langley, 1999; Gordon and Finch, 2005; Yan et al., 2011). There are many advantages of QTL mapping with AA in rice landraces or different germplasm resources, such as (1) Genotypes remain constant from generation to generation; (2) Each phenotype can be observed repeatedly in different environments, which can reduce the measurement errors and environmental effects; (3) Accumulated recombination events can be applied to locate a fine scale QTL; (4) There is no need to test hybrids and their segregating offsprings, and (5) After the identification of QTL location, the effect of each QTL can be estimated by the best linear unbiased prediction (BLUP), and the breeders can select excellent lines in the most convenient way.

## Genetic diversity and genomic variation in rice AA populations

AA cannot be performed in the absence of measurable polymorphisms, so abundant differences at the phenotypic level and a high density of polymorphisms at the DNA sequence level are essential (Yan et al., 2011). Abundant genetic diversity and genomic variation in the rice gene pool improves the mapping power of AA. For instance, a GWAS was carried out in 446 *O. rufipogon* accessions for leaf sheath color and tiller angle, which would have stronger mapping power owing to higher levels of genetic diversity in the wild species than that in *O. sativa* (Huang et al., 2012a). The previous studies found that the genetic diversity of modern cultivars had been reduced compared to the landraces and wild progenitors owing to human and natural selection in rice (Huang et al., 2012a), maize (Hufford et al., 2012), and foxtail millet (Jia et al., 2013), which had been summarized in review of Huang and Han (2014). Based on above studies, the genetic diversity of natural populations in rice AA which were constructed from wild progenitors and landraces might be larger than from modern cultivars. Furthermore, Huang et al. (2012b) and Zhang et al. (2011) found that the genetic diversity of *indica* rice were more abundant than *japonica* rice. And there are studies using only *indica* and *japonica* rice populations for AA where robust trait-marker associations were identified in studies of Lu et al. (2015), Feng et al. (2016), and Yano et al. (2016), respectively. In addition, the genetic diversity and genomic variation as well as mapping resolution of family-based populations might be lower than those in natural populations used in AA due to limited recombination and allele variations. However, the mapping power, especially the power to detect minor effect loci and epistatic interactions within family-based populations might be higher than using natural populations because the genetic backgrounds of family-based populations are much simpler and clearer than natural populations (Wen et al., 2016).

The genomic variations in rice were highly abundant according to the genomic sequences of thousands of rice accessions or cultivars through genotyping by sequencing (GBS) and re-sequencing. High-throughput loci or markers were developed for genotyping the individuals among AA populations based on the genomic variations. The AA methods in model species (i.e., rice, maize, and Arabidopsis) will guide and push forward the development of the other plants AA.

## Population structure and LD in rice AA

Information about the population structure and extent of LD within the population is of fundamental importance for association mapping (Stich et al., 2005). Population structure depends on various factors such as adaptation or domestication and is an important component for association mapping analysis because it can reduce both type I (false positive) and II (false negative) errors between molecular markers and traits of interest in inbreeding species (Goldstein and Weale, 2001; Yu et al., 2006). The presence of subpopulations can result in spurious associations due to confounding of unlinked markers with phenotypic variation (Buckler and Thornsberry, 2002). Genetic loci that do not have any effect on a trait may demonstrate statistical significance for their co-segregations with the trait of interest due to population stratification caused by the genetic drift, domestication, or background selection. The decay of LD over physical distance in a population determines the required marker density and the level of resolution that could be achieved in an association study. If LD decays too fast within a region, then a large number of markers would be required to scan the whole genome or one gene region.

### Population structure in rice AA

The number of identified population structure varied greatly and is summarized in Table 1. There are some studies that were specifically conducted for population structure analysis. Five major groups, i.e., *indica*, aus, aromatic, temperate *japonica*, and tropical *japonica* were detected in a sample of 234 rice varieties (Garris et al., 2005). A similar population was detected by re-sequencing of 50 accessions of cultivated and wild rice accessions (Caicedo et al., 2007; Huang et al., 2012b; Xu et al., 2012). Seven subpopulations were detected within rice landraces (Zhang et al., 2007). Two subgroups, including *indica* and *japonica* as well as six sub-subgroups, were found within a primary rice core collection (Zhang D. et al., 2009). Three subgroups (*japonica*, Aus, and *indica*) were identified within 20 rice varieties/landraces (McNally et al., 2009). Two distinct subgroups (*indica* and *japonica*) were detected within the entire population by different statistical methods, and SG 1 was divided into four sub-subgroups, including intermediate seasonal *indica*, sub-tropical *indica*, late seasonal *indica*, and early seasonal *indica* (Zhang et al., 2011). Three subgroups were detected in a population comprising of 446 wild rice accessions (Huang et al., 2012a). The varied number of subgroups might be due to different methods, different markers number, different rice populations used for population structure analysis, and that needs to be further studied. In general, the subpopulation's information identified in rice population structure studies reflect the history of genetic drift, domestication, or background selection that can effectively reduce the false positive induced by population structure.

When a population structure was assessed by markers or loci, the information regarding subpopulations were considered as covariates in rice AA. For instance, in the study of Famoso et al. (2011), AA was performed within the entire population and all subpopulations, and different significant loci associating with aluminum tolerance were detected in the entire population and sub-populations.

### LD level in rice AA

Marker density is one of the most important factors for an accurate identification of LD level within AA population. Some previous studies suggested that the true LD could be detected by using a modest number of SNPs and SSRs. For instance, Yonemaru et al. (2014) revealed that 20% (1152 SNPs, the marker density is only one marker per 325.10 kb) of 5760 SNPs could detect the LD with high accuracy as that detected by whole markers. Apparently, it is unlikely that a modest number of markers could saturate the whole genome. The marker density ranged from one SNP per 0.06–325.10 kb when rice landraces and diverse collections were used for GWAS of rice. The marker density might be higher than this; however, balanced populations were used for GWAS of rice.

LD varies greatly among different genomic regions and rice populations. Low level of LD would lead to impractical whole-genome scanning because of the excessive number of markers required for whole-genome studies (Kruglyak, 1999). Moreover, the resolution of AA in a population depends on the structure of LD across the whole genome (Remington et al., 2001). The LD decay rate of the population was measured as the chromosomal distance at which the average pairwise correlation coefficient (*r*^2^) dropped to half of its maximum value. Significant LD surrounding the *Xa5* locus of rice was observed between the sites up to 100 kb apart (Garris et al., 2003). LD was observed to decay at 1 cM or less in rice investigated with DNA sequences (Olsen et al., 2006; Mather et al., 2007; Rakshit et al., 2007), while LD decayed at 20–30 cM using SSR markers (Agrama et al., 2007; Agrama and Eizenga, 2008). LD extends to ~200 kb for the *indica* group, but there were only 8 *indica* varieties (McNally et al., 2009). Intra-chromosomal LD decayed at an average of 25–50 cM in different subgroups (Jin et al., 2010). Genome-wide LD decay rates of *indica* and *japonica* were estimated at ~123 and 167 kb, where the *r*^2^ drops to 0.25 and 0.28, respectively (Huang et al., 2010). The LD decay distance was in the region of 40–50 cM in a rice core collection (Zhang et al., 2011). The LD decay was faster in the *indica* subpanel (*r*^2^ below 0.2 at 101 kb) than in the *japonica* subpanel (*r*^2^ below 0.2 at 425 kb; Phung et al., 2014). The minimum distance of LD decay for POP1–POP7 was 60.2, 13.0, 85.4, 70.8, 29.8, 72.9, and 61.8 cM, respectively (Dang et al., 2014). The average LD maximum distance (~125 kb) was observed for chromosomes 8 and 12, while minimum distance (~69 kb) was observed for chromosome 3 (Kumar et al., 2015). These studies suggest that the extent of LD varies greatly among different genomic regions and rice populations. Thus, the marker density used for scanning the whole genome or one gene region depends on the LD decay across the genome or a gene identified in rice by AA.

## Genotyping methods in rice AA

SSRs and SNPs markers have been widely used for rice AA, while amplified fragment length polymorphism (AFLP) markers, restriction fragment length polymorphism (RFLP) markers, inter simple sequence repeat (ISSR), insertion-deletion (InDel), and diversity arrays technology (DArT) markers were not used so frequently (Table 1). For CGAS, AA based haplotype (not based on single SNP locus) might be a good method to find natural allelic variation in traits, and some CGAS studies had used this method (Table 1). For GWAS, higher mapping resolution for AA can be obtained through high marker density, because low marker density considerably reduced the QTL mapping power (Emma et al., 2013). SSR markers have been used for rice genetic maps because of their abundance in rice genome, co-dominance, and a high polymorphism rate (Powell et al., 1996). In the last 10 years, rapid development of the bioinformatics and the completed rice genome has eased the process of SSR markers designing, thus more than 18,830 SSR markers have been developed for rice genome (IRGSP, 2005). Several studies have found low resolution by using SSR markers for AA. With the development of sequencing technology, many complex QTLs or genes have been discovered by re-sequencing the whole genome for AA in the last 4 years (Table 1). SNPs represent the existence of single nucleotide variation in different DNA sequences for a given species. The greatest advantage of SNP is that there are rich polymorphisms in the genome. Genes associated with specific biological traits can be identified using the SNPs marker and LD mapping.

The technique of GBS has recently been successfully utilized for AA studies in maize (Lipka et al., 2013), sorghum (Morris et al., 2013), and wheat (Saintenac et al., 2013). Moreover, GBS is also used for the identification of genes or QTLs that underlie traits of particular interest for breeders such as yield, flowering time as well as plant height, and has been successfully utilized for AA studies in rice (Huang et al., 2012b, 2015; Han and Huang, 2013).

## Phenotyping in rice AA

AA has been proved that it could be an efficient strategy for dissecting many complicated traits in rice. In most of rice AA researches, agronomic and quality traits that link closely with production were often dissected, while biotic stress resistance, abiotic stress resistance and metabolic traits were also studied using AA (Table 1). However, many trait-marker associations proposed to date have not been consistently replicated across different populations in all AA. The percentage of significant associations identical with previously mapped loci ranged from 20 to 75% when SSR markers were used in rice AA. Furthermore, the significant associations identified in rice AA are hard to be repeated in different AA populations (Zhang et al., 2014). While SNP markers were used in rice GWAS, there may be smaller proportions (< 20%) of significant associations identical with previously mapped loci but most of the identical significant loci located at the region of cloned genes. Non-replication often reflects false positives in the original claims and it may due to different parents/populations/markers used in AA as well as heterogeneity caused by biases or even genuine diversity of the genetic effects in different populations (Moonesinghe et al., 2008). However, we think that non-replication in rice AA is likely due to incorrect phenotyping, especially for field phenotyping (agronomic and quality traits of rice are very complicated) in rice AA. Identifying phenotype correctly is one of the key points for a successful AA. For correct identification of phenotype, the assay should be arranged in several years and locations as well as set replications, especially for complex traits.

In addition, high-throughput GWAS corresponding to high-throughput phenotyping could be an extremely effective approach for dissecting complex traits. With high-throughput sequencing techniques rapidly developing, traditional plant phenotyping lags far behind. However, studies on high-throughput phenotyping are being on emergence. For instance, Yang W. et al. (2014) combined high-throughput phenotyping and GWAS to monitor 13 traditional agronomic traits and 2 newly defined traits during the rice growth period. Moreover, the development of multiple omics technology and its combination in AA will be an effective way for dissecting traits such as metabolic traits. For example, Matsuda et al. (2015) performed GWAS to investigate the genetic architecture behind the natural variation of rice secondary metabolites.

Furthermore, agronomic and quality traits as well as rice food safety under fluctuating environment of earth may be the hotspot for future rice AA. It was found that chronic ozone exposure significantly decreased seed weight, culm length, number of primary rachis branch, and number of spikelets per panicle in an *indica* rice Habataki (Tsukahara et al., 2013). Similarly, Ueda et al. (2015) reported a GWAS in rice (*Oryza sativa L*.), which determined candidate loci associated with ozone tolerance.

## Statistical methods in rice AA

Nucleotide diversity measure, discriminant analysis, elliptic fourier analysis, nested clade analysis, principle component analysis, generalized linear model (GLM), MLM, logistic regression (LR), and simple linear model (SLM) were used for rice AA (Table 1). There was no consideration for population structure and relatedness in previous rice AA until Yu et al. (2006) indicated that population structure and relatedness may cause false positives in AA. To overcome the false positive caused by population structure and relatedness, an approach using MLM that takes both population structure (Q) and kinship (K) into account for the reduction of false positives was proposed for association mapping (Yu et al., 2006; Kang et al., 2010; Listgarten et al., 2010; Price et al., 2010; Zhang et al., 2010). In recent years, comparisons of different statistical models e.g., Q, Q+K, and P+K conducted for Arabidopsis (Zhu and Yu, 2009), sweet sorghum (Wang et al., 2009), maize (Yang et al., 2010), and rice (Table 1) indicated that MLM is one of the most appropriate and popular methods used for AA. However, MLM should be improved for the detection of rare alleles.

It is important to point out more explicitly that GWAS is likely to identify associations of common alleles, but are not suitable for the analysis of rare alleles/haplotypes due to statistical significance issues unless the use of specifically designed balanced population. Almost all of the statistical methods used for rice AA could filter the variants or loci with minor allele frequency (< 0.05), and this leads to missing of some functional rare alleles. Therefore, a new statistical method, called as Anderson-Darling (A-D) test, which could control rare alleles in GWAS, was reported. Moreover, the A-D test is one of the useful complement for GWAS analysis of complex quantitative traits in rice AA. The A-D test balances the false positives and statistical power (Yang N. et al., 2014).

The statistical level (*P*-value) cut-off for significance in rice AA ranged from 10^−8^ to 0.05 (Table 1). The most stringent significant level was *P* < 10^−4^ (Wen et al., 2009) for AA in rice until SNP markers were reported in the study of Huang et al. (2010). Moreover, the significant level was set more stringent than *P* < 10^−4^ when SNP markers were used in rice AA (Table 1). In general, the more stringent significant level is set, the less false positives will be identified, while the more false negatives will be created. However, setting less stringent significant level may be due to the lower marker density when using limited number of SSR markers. Therefore, the significant trait-marker associations identified under less stringent significant level might be true significant associations. For instance, the percentage of significant associations identical with the previously mapped loci were 68% (Agrama et al., 2007) and 75% (Wen et al., 2009) corresponding to 0.05 as well as 10^−4^ of *P*-value, respectively. We conclude that the statistical significance level should be set according to the marker density used for AA in rice. The higher the marker density, the more stringent significant level should be set.

## Proven benefits of rice AA

Since rice landraces with abundant natural variations were first used in AA, especially in GWAS, the approach has revealed that it is an efficient strategy for dissecting many complicated traits in rice. For instance, in the GWAS study of Huang et al. (2010), association signals for apiculus color, pericarp color, gelatinization temperature, amylose content, grain width, and grain length were located close to known genes that have previously been identified using mutants or recombinant populations in studies of Saitoh et al. (2004), Sweeney et al. (2006), Gao et al. (2003), Wang et al. (1995), Shomura et al. (2008), and Fan et al. (2006), respectively.

In most of the rice AA studies, agronomic and qualitative traits that tightly linked with production/yield were dissected (Table 1), resistance to biotic stress (Zhao et al., 2011; Jia et al., 2012; Wang C. et al., 2014), abiotic stress (Ahmadi et al., 2011; Famoso et al., 2011; Norton et al., 2014; Kumar et al., 2015; Ueda et al., 2015), and metabolic traits were also studied using AA (Chen et al., 2014; Matsuda et al., 2015). Large-scale AA (GWAS) has led to the discovery of thousands of genetic signals across the rice genome associated with plant quantitative traits.

## Major shortcomings and possible solutions in rice AA

**Figure 1:**
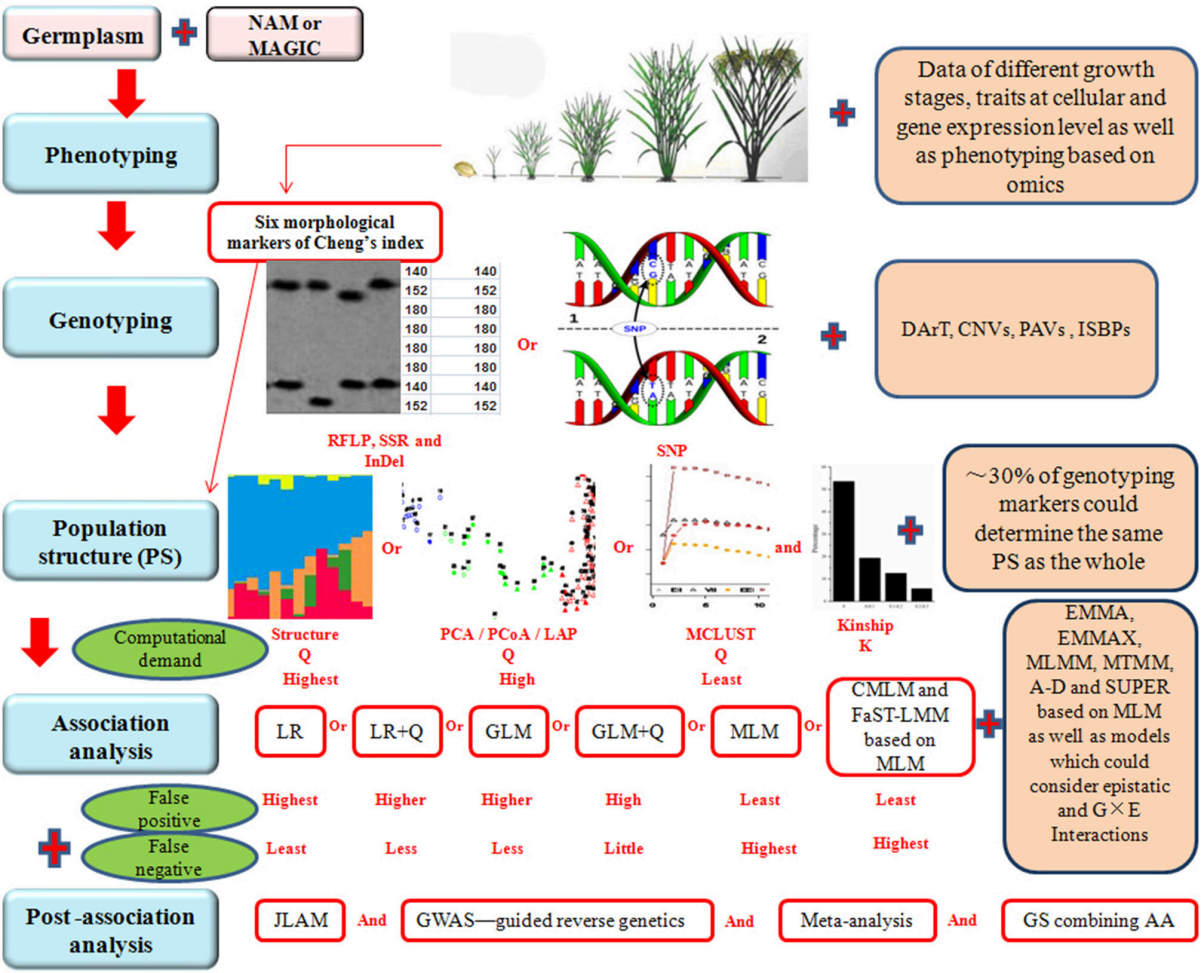
**Schematic representation of various steps involved in association analysis (AA)**. NAM, nested association mapping. MAGIC, multi-parent advanced generation intercross. RFLP, restriction fragment length polymorphism. SSR, simple sequence repeat. InDel, insertion-deletion. SNP, single nucleotide polymorphisms. DArT, diversity array technology. CNVs, copy number variations. PAVs, presence and absence variations. ISBPs, insertion-site-based polymorphisms. PCA, principal component analysis. PCoA, principal coordinate analysis. LAP, laplacian eigenfunctions. LR, logistic regression. GLM, generalized linear model. MLM, mixed linear model. CMLM, compressed mixed linear model. FaST-LMM, factored spectrally transformed linear mixed model. EMMA, efficient mixed model association. EMMAX, EMMA eXpedited. MLMM, multi-locus mixed model. MTMM, multi-trait mixed model. A-D, Anderson-Darling test. SUPER, settlement of MLM under progressively exclusive relationship. G, genotype. E, environment. JLAM, joint linkage association mapping. GWAS, genome wide association study. GS, genomic selection. Plus sign (Red) represents the performance which should be improved in future rice association analysis.

Although AA is an effective way to construct fine maps for quantitative traits, there are some problems to be solved in the future to improve the efficiency of AA. For instance, the results of AA studies are hardly being used in rice breeding. Gupta et al. (2014) indicated that underutilization in plant breeding is partly due to high false discovery rate (FDR), and partly due to the difficulty in using markers with rare alleles that may be associated with missing and desirable heritability for the traits of interest. These problems and possible solutions are discussed below in detail and a schematic presentation showing the problems and possible solutions in each steps during AA is given in Figure 1.

There is no previous report about the existence of false positives existed in rice AA because the focus of rice AA is only to detect significant trait-loci, and few studies have conducted follow-up tests of rice AA candidate genes. False positives in rice AA are possible due to linkage, population structure, familial relatedness, and low repeatability.

### The false positives due to linkage

Linkage between causal and non-causal sites as well as epistasis can induce false positives and true genes or loci cannot be identified because very large linkage complexes are hard to resolve into small fragments. This problem has been demonstrated by the two studies: Dickson et al. (2010) used simulations to demonstrate the presence of two or more rare causal variants in disequilibrium that cannot be detected due to the lack of statistical power and can produce spurious associations that are distantly linked to the causal polymorphisms; and Atwell et al. (2010) revealed that negative disequilibrium between two causal polymorphisms in the gene *FRIGIDA* interfered with the ability to find either of them but created strong signals at several distantly linked markers in a genome-wide association study in *Arabidopsis thaliana*. These false positives caused by linkage cannot be eliminated by increasing the sample size or number of markers and this problem was always found in pleiotropy (Platt et al., 2010). However, constructing the haplotypes with multiple traits by GBS might be a possible way to reduce the rate of false positives by linkage. For instance, Larsson et al. (2013) and Morris et al. (2013) have shown that association and linkage mapping combined with haplotype diversity can produce more robust results.

### The false positives due to population structure and familial relatedness

The presence of subpopulations and kinship can result in spurious associations due to confounding of unlinked markers with phenotypic variation (Buckler and Thornsberry, 2002). For instance, *Dwarf 8* associations reported by Thornsberry et al. (2001) was generally accepted as the first plant association mapping study, but these associations are likely false positives which resulted from insufficient correction of population structure (Larsson et al., 2013).

Scientists have always tried to control the effects of population structure and kinship in AA studies. Three statistical methods (i.e., genomic control, structured association method, and the unified mixed model approach) are often recommended to control the effects of population structure in association studies, and these three methods are well-summarized in review of Gupta et al. (2014). However, population structure identified by Laplacian Eigen functions (LAP; Zhang J. et al., 2009) and MCLUST (Fraley and Raftery, 2007) as well as morphological markers were not described by Gupta et al. (2014). Furthermore, the Cheng's index method could discriminate between *indica* and *japonica* rice cultivars based on six morphological traits, i.e., hair, phenol reaction, length of 1st–2nd rachis internode, glume color at heading, leaf hair, and grain length/width ratio (Xu et al., 2005; Zhu et al., 2007).

Moreover, the population structure identified by principal component analysis (PCA), principal coordinate analysis (PCoA), and LAP is highly related to the known germplasm type information as well as the STRUCTURE subgroups (Zhang et al., 2011). These methods have neither computation burden nor assuming any population genetic model. However, they don't provide the information on the number of subgroups and assignment of individuals to subgroups. STRUCTURE and MCLUST could provide the information of subgroups by a detail membership probabilities threshold that could be used as covariant in AA. Population structure was constructed by using the morphological traits and the data of both morphological and molecular markers was also compared, and it was found that MCLUST based on the morphological markers might be the cheapest method to detect population structure (Zhang et al., 2011).

MLM is one of the most popular methods for controlling population structure and familial relatedness in rice AA. However, Segura et al. (2012) showed that MLM could not always account for a locus with larger effects. Furthermore, Zhou and Stephens (2012) showed that existing methods for exact computation of standard statistical tests were computationally impractical for even moderate-sized genome-wide association studies. The consensus until now has been that all available SNPs should be used to determine population structure and familial relatedness using MLM (Listgarten et al., 2012). Therefore, MLM's intensive computational burden is prohibitive in practice, especially for large samples (Wang Q. et al., 2014). To date, a few strategies have been used to improve MLM. The newly developed algorithm, FaST-LMM, solved the computational problem, but requires fewer number of SNPs than the number of individuals to derive a rank-reduced relationship (Lippert et al., 2011). This restriction potentially leads to less statistical power compared to using all SNPs. A small number of SNPs (called factored spectrally transformed linear mixed model, FaST-LMM-Select) systematically increase power, improve calibration and reduce computational cost to structured populations (Listgarten et al., 2012). Moreover, an efficient method that was named as genome-wide efficient mixed-model association (GEMMA) was presented, which makes approximations unnecessary in many contexts (Zhou and Stephens, 2012). A multi-locus mixed model as a general method was proposed for mapping complex traits in structured populations (Segura et al., 2012). Wang Q. et al. (2014) developed a SUPER (Settlement of MLM Under Progressively Exclusive Relationship) powerful method that dramatically reduces the number of genetic markers in defining individual relationships and remarkably increases statistical power.

Computational burden is mainly due to the use of too many markers for GWAS. All the above strategies which require fewer markers could improve MLM computational efficiency and identify population structure and individual relatedness correctly by newly developed algorithms. Meanwhile, in our previous study (Zhang et al., 2011), studies of Li J. et al. (2011) and Van Inghelandt et al. (2010), 25~30% of all markers were required to determine the same population structure as the whole markers, and similar precision was found in the population by both numbers of markers. The ideal results may be achieved with MLM if a small number of markers (e.g., hundreds or thousands of markers) will be used for AA, while FaST-LMM, GEMMA, MLMM, and SUPER should be used, especially for complex traits, if a huge number of markers (e.g., millions of markers) are available for AA.

### The false negatives due to overcompensation

False negatives (missed significant signals) caused by overcompensating corrections of multiple testing for significant associations and problems with rare alleles.

There are many corrections that have been suggested to overcome the problem of false positive and negative associations due to multiple testing and some of the commonly used corrections are as follow: (1) Bonferroni correction (Moran, 2003); (2) Holm correction (Holm, 1979); (3) FDR (Benjamini and Hochberg, 1995); 4) *q*-value (Storey, 2002); and (5) step-up adaptive method (Benjamini et al., 2006). Gupta et al. (2014) has explained these five corrections in detail and made an excellent comparison of statistical methods used for corrections to overcome the multiple testing problems (genome-wide error rate and FDR). They concluded that the reasonable choice of corrections would be to compare the results obtained by using different methods, and to evaluate the differences in the number of QTLs identified with biological significance. We fully agree with the above mentioned conclusions of Gupta et al. (2014).

Furthermore, GWAS has low power for rare alleles, which makes a substantial proportion of natural variations. The sites with weaker effects may play an important role in evaluation of traits like the sites with stronger effects. Some studies about the detection of rare alleles have been performed. For instance, several statistical models for rare alleles in AA have been summarized (Gibson, 2011). Moreover, Sur et al. (2013) indicated that the next step in the genetic epidemiology of breast cancer needed to include the assessment of variants with lower frequencies and smaller effect sizes.

About 44% of the SNPs are of low frequency (minor allele frequency <0.05) in rice. It has been concluded that the use of a large sample size or the construction of multiple bi-parental cross populations (e.g., NAM or MAGIC) may be helpful in rice GWAS for rare alleles (Huang and Han, 2014). To detect rare marker alleles from the analysis, linkage mapping and LD mapping could be combined for conducting joint-linkage association mapping (JLAM; Gupta et al., 2014).

### Computational capacity for identifying population structure

A dataset with large sample size and plenty of markers creates a demand for heavy computational capacity in the MLM approach (compared with GLM; Zhang et al., 2010). We have summarized the methods to improve the computational capacity based on previous and emerging studies in the section entitled “*The False Positives due to Population Structure and Familial Relatedness*” of this review and have also been discussed by Gupta et al. (2014). However, all of the above improvements are performed in the process of trait-marker association, which might be a more efficient way to reduce the computational demand before the association is done. For instance, identifying population structure, especially using software such as STRUCTURE, also creates a heavy computational demand.

The consensus until now has been that all available SNPs should be used to determine population structure and familial relatedness using MLM (Listgarten et al., 2012). However, using 359 SSRs and 8244 SNPs for detecting the population structure of 1537 maize accessions, Van Inghelandt et al. (2010) showed that the population structure was consistent based on SSRs and SNPs. Furthermore, SSR markers have their own advantages as compared to SNP markers with respect to population genetics (Van Inghelandt et al., 2010). Abundant SSR and SNP markers can provide an important technology to support further research on LD structure, gene fine mapping and association analysis of crop germplasm resources. Furthermore, in our previous study, Zhang et al. (2011) indicated that about 72 SSR markers (26% of the total markers) were required to determine the same population structure as the whole 274 SSR markers, and a similar precision was found in the rice core collection using both numbers of markers. These results were consistent with the research of: (1) Li J. et al. (2011), who reported that 100 out of 328 SNPs (30%) were required to examine the population structure of sugar beet with similar accuracy as detected by the whole data set; (2) Van Inghelandt et al. (2010), who revealed that 25% of the SSRs (90 out of 359 SSRs) could detect the population structure with similar accuracy as the whole SSR markers did by modified Rogers distance (MRD) estimates. Moreover, we recommend that population structure identified with MCLUST based on the morphological markers may be a convenient method to reduce the computational demand before AA is undertaken—a conclusion indicated in our previous study (Zhang et al., 2011).

### Low repeatability and underutilization of AA

Large-scale GWAS has led to the discovery of thousands of genetic signals across the rice genome associated with rice quantitative traits (Table 1). However, as GWAS is a relatively new approach, there are few studies that have conducted follow-up tests of candidate genes. Furthermore, both the results of linkage mapping and association mapping are hard to repeat due to different parents/populations/markers used in linkage or association mapping. Moreover, there are hardly any documented examples where results of AA studies have been used in rice breeding, though some undocumented examples may be available. This is partly due to the high FDR (Gupta et al., 2014).

To improve repeatability, there is a requirement to carefully perform each step of rice AA as follows: (1) choose large sampling populations. Large sampling populations may contain more genetic recombinations and could be helpful in reducing false negatives (Huang and Han, 2014). We suggest that one convenient way to increase recombinations is by selecting large populations and to construct a core collection by the weighted pair-group average method combined with stepwise clustering with preferred sampling based on taxonomic, geographical, morphological, and agronomic data. This method was reported in detail in the previous research of Li X. L. et al. (2011). Another method is to develop NAM population by crossing a diverse range of landraces or varieties from diverse populations or core collections with one common and well-characterized parent, but it requires time. It is not always “the more, the better” when sampling (especially in plant samples), because the diversity and the individual relationship can greatly increase the population stratification and that may have a strong influence on GWAS (Han and Huang, 2013). The population size used for rice AA ranged from 50 to 1495 accessions in previous studies (Table 1); (2) genotyping of populations. Besides markers like RFLP, SSRs, InDels, SNPs, based on next generation sequencing (NGS), GBS, and DArT used in rice AA (Table 1), other newer approaches can also be designed using copy number variations (CNVs), presence and absence variations (PAVs), and insertion-site-based polymorphisms (ISBPs), which are now being discovered in a number of crops (Edwards and Gupta, 2013); (3) phenotypic measurement of populations. First, the identification of traits by multi-year and multi-locus can efficiently reduce the effect of environment and genetic background (Vilhjalmsson and Nordborg, 2013). In most of the rice AA studies, agronomic and qualitative traits were dissected by multi-year. Second, Gupta et al. (2014) indicated that the popular AA model involving MLM association of a single locus with a single trait leads to misspecification and leads to biased results—we fully agree with this statement. To solve the above problem, the model combining all traits as cofactors, called multi-trait mixed model (MTMM) has been developed in the study of Korte et al. (2012) and has been used in maize (Liu et al., 2013). Furthermore, an approach using MLM, especially for complex quantitative traits, controlled by major effect loci and with normal phenotype distribution (Yang N. et al., 2014), was proposed for association mapping. In most of the rice AA studies, the most popular AA model is MLM. However, there are some other models used in rice AA. For instance, LR, SLM, FaST-LMM, and compressed mixed linear model (CMLM; Table 1). Thirdly, Gupta et al. (2014) also indicated that several developmental traits such as plant height are dynamic in nature, and any two genotypes may have the same plant height but different growth trajectories during development. For this purpose, the data recorded at different developmental stages may be used either independently (data for the same stage) or jointly (data for different stages together). The developmental traits which are dynamic in nature have been detected in rice AA.

How best to utilize the results of rice AA? Many strategies have been suggested to conquer the limitations which have plagued AA are outlined in the review of Gupta et al. (2014). Furthermore, there is a need to undertake efforts to better utilize AA results: (1) identify true significant trait-marker associations, especially for GWAS and the function of candidate genes or loci. GWAS has led to the discovery of thousands of genetic signals across the plant genome associated with plant quantitative traits. However, there might be plenty of false positives. Therefore, more studies should be conducted to test candidate genes or loci detected by AA. Firstly, one effective way is GWAS—guided reverse genetics. The combination of GWAS and reverse genetics can be used to identify new genes efficiently, especially applicable for complex traits that are difficult to analyze by other genetic screening methods. For instance, T-DNA mutants were used to explore regions with strong significant SNPs which were identified with GWAS by Verslues et al. (2014), who identified several new proline effector genes. Moreover, other reverse genetics, like genome editing using transcription activator-like effector nucleases (TALENs) and CRISPR/Cas9 systems may be an effective method for testing candidate genes. Secondly, meta-analysis combining information from AA. We could detect loci near the genes with known functions through meta-analysis, and these loci may be good candidates as functionally relevant genes (Sur et al., 2013). Numerous studies involving GWAS meta-analysis have been published for humans (Evangelou and Ioannidis, 2013). Thirdly, subsequent CGAS based on the results of GWAS may be an efficient way to dissect the function of candidate genes or loci. Fourthly, false positives may not be avoided through the aforementioned models. To avoid them, it is necessary to make sure that the significant associations identified within one population should be present in another population (Wray et al., 2013). There is only one study undertaking AA in rice that has verified significant associations of a panel (population) in two other panels (Zhang et al., 2014); (2) combinations of genomic selection (GS) and AA could also be used to avoid false positives. GS is a new breeding method in which genome-wide markers are used to predict the breeding value of individuals in a breeding population. GS has significantly improved the breeding efficiency in dairy cattle (Hayes et al., 2009) and several crop plant species (Heffner et al., 2009). A combination of GS and GWAS in rice breeding program at the International Rice Research Institute (Philippines) has shown that GS can result in more accurate predictions of breeding line performance than pedigree data alone, and GWAS results can facilitate the results of GS (Spindel et al., 2015); (3) construction of a high quality haplotype map. A genome-wide haplotype map of SNP variation will accelerate molecular breeding by expanding the diversity of germplasm accessible to crop improvement programs and will increase the resolution of GWAS, marker-assisted selection and GS (Morrell et al., 2011). High quality haplotype map can provide an extinct demonstration about varieties corresponding to elite alleles which could be used as the donor of elite genes in crop breeding. Moreover, functional markers can be designed for mining the elite varieties through haplotype map.

## Conclusions

### Prospects of rice AA

#### Population for rice AA

AA is a complementary of linkage mapping, and fine mapping or map-based cloning, and it can give a definite dissection for loci or genes through linkage that have shown significant effect within AA. NAM is another strategy for mapping, which based on both linkage and AA (Yu et al., 2008). Both family-based population and JLAM had proved that they could improve the power of AA, but only two studies have used JLAM approach (Hu et al., 2010; Famoso et al., 2011), while no one has used family-based population for rice AA. We strongly recommend that family-based population (e.g., NAM or MAGIC) and JLAM should be included in future rice AA. Our research group has developed two rice NAM populations, consisting of 15 RILs that were generated by crossing a diverse range of 15 Ting's core collection (landraces) with Nipponbare and 93-11.

#### Phenotyping in rice AA

Identification of a correct phenotype is one of the key points for a successful AA. High-throughput GWAS corresponded to high-throughput phenotyping, which can be an extremely effective approach for dissecting complex traits. With the rapid development of high-throughput sequencing techniques, traditional plant phenotyping lags are far behind. Studies on high-throughput phenotyping are rapidly emerging.

Furthermore, traits at cellular and gene expression level as well as traits based on omics may be one of the focuses in rice AA. For instance, Meijon et al. (2014) used the model organism *Arabidopsis thaliana* to combine high-throughput confocal microscopy imaging of traits at the cellular level, GWAS and expression analyses to identify genomic regions that are associated with developmental cell-type traits. Dick et al. (2014) performed GWAS between methylation levels and body-mass index (BMI) and found that increased BMI in adults of European origin is associated with increased methylation at the ***HIF3A*** locus in blood cells and in adipose tissue. Therefore, phenotyping or phenomics integrating with omics such as genomics, proteomics, metabolomics, transcriptomics, lipidomics, immunomics, glycomics, RNomics will be more useful for dissecting complicated traits in rice or other species AA.

#### Epistatic and genotype (G) × environment (E) interactions

Many important agronomic traits of crops, such as yield and its related traits, plant type, growth period, and resistance to biotic and abiotic stresses, are all complex quantitative traits. It is hard to investigate these characters because of their polygenic control, interactions of multiple loci and effect of environment. In the past few years, the establishment of new statistical methods has enabled us to explore the epistatic interactions between loci and LD between related loci caused by epistatic interactions—this offers new insights to study the epistatic effect and G × E interaction. Gupta et al. (2014) have discussed in detail the necessity and the way QTL interactions (epistasis and G × E) are involved in AA. Moreover, existing models that could consider epistasis and G × E as cofactors should be improved for future rice AA. To date, there is no study about rice AA that has discussed epistasis and G × E.

#### Post-rice AA

Large-scale AA (GWAS) have led to the discovery of thousands of genetic signals across the rice genome associated with plant quantitative traits. However, as GWAS is a relatively new approach, there are few studies that have conducted follow-up tests of candidate genes. It is time to slow down the pace of GWAS and think about how we can conduct analyses post-GWAS. Our opinion about post-GWAS is as follow: we should concentrate on the identification of true significant trait-marker associations, especially those loci that have not been detected in previous linkage mapping, and dissecting the function of candidate genes or loci by using reverse genetics and bioinformatic tools, including meta-analysis as well as a combination of CGAS and GWAS. Si et al. (2016) identified that *OsSPL13* controls grain size in cultivated rice using their previous GWAS results, which gives a direct example of post-GWAS research.

## Author contributions

This review was conceived by PZ, KZ, and HT, the manuscript was drafted by PZ, KZ, MS, and HT. HT acted as a co-corresponding author. All authors read and approved of the final manuscript.

### Conflict of interest statement

The authors declare that the research was conducted in the absence of any commercial or financial relationships that could be construed as a potential conflict of interest.

## Abbreviations

LD: Linkage disequilibrium
QTL: Quantitative trait loci
AA: Association analysis
GWAS: Genome-wide association study
CGAS: Candidate-gene association study
SNPs: Single-nucleotide polymorphisms
MLM: Mixed linear model
BLUP: Best linear unbiased prediction
USDA: United States Department of Agriculture
SSR: Simple sequence repeat
*r*^2^: Pairwise correlation coefficient
RFLP: Restriction fragment length polymorphism
InDel: Insertion-deletion
GBS: Genotyping by sequencing
GLM: Generalized linear model
Q: Population structure
K: Kinship
LAP: Laplacian eigen functions
GEMMA: Genome-wide efficient mixed-model association
SUPER: Settlement of MLM under progressively exclusive relationship
A-D: Anderson-Darling
NAM: Nested association mapping
RIL: Recombinant inbred lines
MAGIC: Multi-parent advanced generation intercross
FDR: False discovery rate
MRD: Modified Rogers distance
NGS: Next generation sequencing
DArT: Diversity array technology
CNVs: Copy number variations
PAVs: Presence and absence variations
ISBPs: Insertion-site-based polymorphisms
MTMM: Multi-trait mixed model
TALENs: Transcription activator-like effector nucleases
CRISPR: Clustered regularly interspersed short palindromic repeats
GS: Genomic selection
BMI: Body-mass index
JLAM: Joint-linkage association mapping
LR: Logistic regression
SLM: Simple linear model
FaST-LMM: Factored spectrally transformed linear mixed model
CMLM: Compressed mixed linear model
EMMA: Efficient mixed-model association
EMMAX: Efficient mixed-model association eXpedited.
